# Combination of Fresh Frozen Plasma and Cryosupernatant Plasma for Therapeutic Plasma Exchange in Thrombotic Thrombocytopenic Purpura: A Single Institution Experience

**DOI:** 10.1155/2019/1756109

**Published:** 2019-01-30

**Authors:** Qiuyan Lin, Liping Fan, Haobo Huang, Feng Zeng, Danhui Fu, Shijin Wei

**Affiliations:** ^1^Department of Blood Transfusion, Fujian Medical University Union Hospital, Gulou District, Fuzhou City, Fujian Province 350001, China; ^2^Department of Hematology, Fujian Medical University Union Hospital and Fujian Institute of Hematology, Gulou District, Fuzhou City, Fujian Province 350001, China

## Abstract

**Purpose:**

To evaluate the impact of a combination of fresh frozen plasma (FFP) and cryosupernatant plasma (CP) as a replacement fluid in therapeutic plasma exchange (TPE) on early therapeutic response and long-term survival of patients with thrombotic thrombocytopenic purpura (TTP).

**Materials and Methods:**

A total of 44 patients with suspected TTP were screened by Bentley and PLASMIC scores. Twenty-seven patients treated with TPE using the FFP and CP combination as the replacement fluid were enrolled and divided into two groups: 11 patients who received TPE with CP-dominant replacement fluid (FFP/CP<1) and 16 patients who received TPE with FFP-dominant replacement fluid (FFP/CP>1).

**Results:**

There were no significant differences in the demographic and clinicopathological characteristics between the two groups except for the international normalized ratio (INR). The number of TPE procedures was lower, and time to achieve complete response was shorter in the CP-dominant group than in the FFP-dominant group. There were no significant differences in overall survival between the two groups.

**Conclusion:**

The CP-dominant replacement fluid was superior to the FFP-dominant replacement fluid in early response to TPE in patients with TTP, but did not impact the patients' overall survival.

## 1. Introduction

Thrombotic thrombocytopenic purpura (TTP) is a life-threatening form of thrombotic microangiopathy, characterized by microangiopathic hemolytic anemia (MAHA), thrombocytopenia, and neurological deficits, renal injury, and fever caused by the severe deficiency of plasma ADAMTS13 activity. Although new drugs in recent years show promise in the management of TTP, therapeutic plasma exchange (TPE) remains the frontline treatment for TTP. Replacement fluids of TPE provide exogenous ADAMTS13, which reduces the generation of abnormal high-molecular-weight vWF multimers and prevents the generation of platelet rich-thrombi [1–3]. Basic studies have demonstrated that fresh frozen plasma (FFP), plasma virally attenuated with solvent/detergent (SD-FFP), and cryosupernatant plasma (CP) contain equivalent ADAMTS13 activity [1, 4]. Several clinical studies using the replacement fluids mentioned previously showed that TPE using CP had an impact as a single replacement fluid on early therapeutic response and long-term survival of patients with TTP and was inferior to those using FFP or SD-FFP [5–9]. Due to the pathogenesis of TTP, the selection of replacement fluid may be an essential factor related to the therapeutic response for patients with TTP. To date, there have been no studies investigating the impact of a FFP and CP combination as a replacement fluid in TPE on patients with TTP.

Here, we reviewed the medical records and follow-up data of patients with TTP at our institution. These data provide more information for improving replacement fluid selection in TPE treatments of patients with TTP.

## 2. Materials and Methods

### 2.1. Ethics Statement

This study was approved by the Ethics Committee of Fujian Medical University Union Hospital. As this study was retrospective and did not affect the patients' treatments, written informed consent from patients was not sought.

### 2.2. Patients

Patients diagnosed with suspected TTP and receiving TPE treatment immediately between January 2013 and March 2018 were included in this study. The Bentley score [10] was used to exclude the diagnosis of TTP in all patients included. The PLASMIC score [11, 12] was used to predict the severity of ADAMTS13 activity deficiency in all patients included. Demographic and clinicopathological characteristics were reviewed. The patients were followed up from January 2013 to August 2018.

Patients who received the TPE treatment in less than two procedures, those without all five components of the Bentley score, or those with Bentley scores less than 20 points were excluded. Plasma ADAMTS13 activity was detected in some of the enrolled patients.

According to ratio of FFP to CP used per patient, patients were divided into two groups: the CP-dominant group (FFP/CP<1) and FFP-dominant group (FFP/CP*⩾*1).

### 2.3. Methods

The TPEs were performed by trained apheresis technologists with blood cell separators (COBE Spectra, Terumo BCT; MCS+, Haemonetics). Plasma products acting as replacement fluids were supplied by the Fujian Province Blood Center. The TPE procedures were performed with 1–1.5 volumes of plasma daily until complete therapeutic response was reached. After that, the TPE procedures were performed with 1–1.5 volumes of plasma every other day. The decision to cease TPE was made by clinical physicians.

Steroids (1 mg/kg/day) were used for 3 weeks as a combined treatment to TPE in patients with newly diagnosed TTP. Vincristine (1.4 mg/m^2^, up to 2.0 mg total dose), cyclophosphamide (0.4 g once a week, up to 2 g total dose), intravenous immunoglobulin (IVIg) (20 g/d, for 3-5 days), or rituximab (600 mg once a week, for 2-4 weeks) was used as an alternative treatment when patients achieved suboptimal response to treatments combining TPE with steroids.

Blood samples were acquired before suspected TTP patients received TPE treatment. Detection of plasma ADAMTS13 activity was performed at Beijing Hightrust Diagnostic Medical Test Laboratory using the surface-enhanced laser-desorption/ionization time-of-flight mass spectrometry.

Complete responses to treatment, remission, exacerbation, and relapse were defined according to the classification, diagnosis, and management of TTP from 2012 American Society for Apheresis (ASFA) consensus conference [13].

The time to achieve complete response was measured from the date when TPE began to the date the patient achieved the standard of complete response. Overall survival was measured from the date on which the patient was hospitalized to the date of death or last follow-up. All causes of death were included. Survival times were measured until August 22, 2018.

### 2.4. Statistical Analysis

All statistical analyses were performed using the SPSS 19.0 software for Windows. The Chi-square test and independent* t*-test were used to analyze categorical and continuous variables of patients between the CP-dominant group and FFP-dominant group, respectively. The Kaplan-Meier method was used for calculating overall survival, and the log-rank test was used for analyzing the differences among these survival curves. Two-sided* P* values of <0.05 were considered statistically significant.

## 3. Results

### 3.1. Patient Demographic and Clinicopathological Characteristics

**Figure 1:**
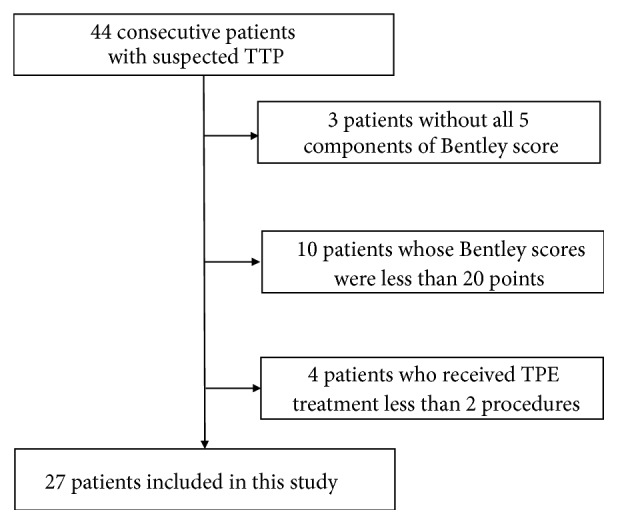
Patient selection criteria for this study.

A total of 44 patients with suspected TTP were identified from January 2013 to March 2018. A total of 17 patients were excluded: 3 patients without all five components of the Bentley score, 10 patients whose Bentley scores were less than 20 points, and 4 patients who received TPE treatments in less than two procedures. The remaining 27 patients were enrolled in this study (Figure 1). Demographic and clinicopathological characteristics, components of PLASMIC, and Bentley scores of the patients enrolled in this study are listed in Tables 1 and 2.

### 3.2. PLASMIC Score and ADAMTS13 Activity of Patients in This Study

All patients enrolled had no previous history of transplantation. None of the patients had low PLASMIC scores (0–4), one patient had intermediate PLASMIC scores (5), and 26 patients had high PLASMIC scores (6–7). Only five patients were assessed for ADAMTS13 activity, which was less than 5% (0-2.5%) for all patients, and their PLASMIC scores were all greater than 6 (Table 2).

### 3.3. Number of TPE Procedures That the Patients Received in This Study

Patients No. 3, 6, and 18 received 6, 6, and 4 TPE procedures, respectively, and did not achieve complete response before the clinical physicians ceased TPE for unknown reasons. Except for the three patients previously mentioned, the number of TPE procedures performed in patients of this study ranged from 2 to 23, with a mean of 6.8 TPE procedures per patient. In the CP-dominant group, 11 patients received 47 TPE procedures, with a mean of 4.3 TPE procedures per patient. In the FFP-dominant group, 13 patients received 117 TPE procedures, with a mean of 9 TPE procedures per patient.

### 3.4. Number of TPE Procedures and Time to Achieve Complete Response

Except for the three patients (No. 3, 6, and 18) previously mentioned, the number of TPE procedures performed to achieve complete response in patients of this study ranged from 2 to 23, with a mean of 6.3 TPE procedures per patient. In the CP-dominant group, 11 patients received 44 TPE procedures, with a mean of 4.0 TPE procedures per patient. In the FFP-dominant group, 13 patients received 107 TPE procedures, with a mean of 8.2 TPE procedures per patient. There was a significant difference in the number of TPE procedures to achieve complete response in the patients between CP-dominant group and FFP-dominant group (4.00 ± 0.92 vs 8.23 ± 1.63, P=0.04) (Table 1).

Except for the three patients (No. 3, 6, and 18) previously mentioned, the time to achieve complete response in the patients of this study ranged from 3 to 47 days, with a mean of 10.3 days per patient. There was a significant difference in time to achieve complete response in patients between the CP-dominant group and FFP-dominant group (5.82 ± 1.39 days vs 14.08 ± 3.32 days, P=0.04) (Table 1).

### 3.5. Other Therapeutic Methods Used in Patients

In addition to TPE, other therapeutics, such as steroids, vincristine, cyclophosphamide, IVIg, and rituximab, were used for patients with TTP in our study. There were no significant differences due to the use of other therapeutic methods used for patients with TTP between the CP-dominant group and FFP-dominant group (Table 1).

### 3.6. Overall Survival of Patients in This Study

**Figure 2:**
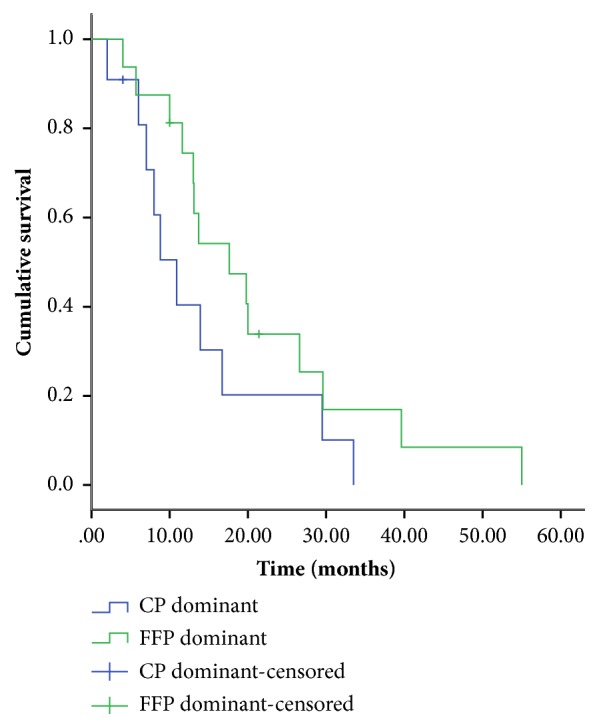
Kaplan-Meier curve of overall survival of patients with thrombotic thrombocytopenic purpura treated with therapeutic plasma exchange with fresh frozen plasma (FFP)- or cryosupernatant plasma (CP)-dominant replacement fluids.

All 27 patients were enrolled for the overall survival analysis. During the follow-up period, three patients were lost prior to the follow-up, seven patients died, and 17 patients survived. There were no significant differences in the overall survival of patients between the CP-dominant group and FFP-dominant group (13.75 ± 3.25 months vs 21.36 ± 3.84 months, P=0.14) (Figure 2).

## 4. Discussion

TTP is a rare hematologic disorder. The pathogenesis of TTP is based on unrestrained growth of microvascular platelet rich-thrombi due to the deficiency of plasma ADAMTS13 activity, the only biologic marker specific for TTP [1–3]. Therefore, the detection of plasma ADAMTS13 activity is important for the diagnosis of TTP. The limited methodologies available for detecting ADAMTS13 activity make it almost impossible to obtain reliable results of ADAMTS13 activity in an emergency [3]. Therefore, some researchers have established point-scoring systems to predict deficiencies of ADAMTS13 activity and prognosis of TTP [10–12].

In our study, we used the Bentley score to predict the prognosis of TTP, and the PLASMIC score to predict the severity of ADAMTS13 deficiency. The Bentley score of all patients enrolled in our study was greater than 20 points. The PLASMIC score of all patients enrolled in our study ranged from 5 to 7. These results are different from those of other studies. We believe that screening using the Bentley score excluded patients who have similar symptoms to TTP but with a different diagnosis. In our study, ADAMTS13 activity was measured in only five patients, revealing severe deficiency of ADAMTS13 activity in the plasma. The PLASMIC scores of these patients were high (6–7). This phenomenon validated the PLASMIC score prediction in the severity of ADAMTS13 activity deficiency, though this evidence is not strong. This result was also similar to that reported in studies from other centers [11, 14, 15].

As the first-line treatment, TPE can replenish ADAMTS13 and block microvascular thrombosis [1–3]. It has been reported that several types of plasma products could be used as the replacement fluid in TPE for patients with TTP [5–9, 16–19]. CP lacking vWF contains an equivalent amount of ADAMTS13 to FFP or its derivative and is considered a better choice than other plasma products, such as FFP, for TPE in patients with TTP [1–3]. However, different studies have shown different impacts of CP as a replacement fluid on the prognosis of patients with TTP, compared with FFP [5–9]. Based on these inconsistent studies, we took the advantages and disadvantages of CP and FFP into account and used a CP and FFP combination for the replacement fluid at our institute. After screening the enrollment criteria, we collected the medical records of 27 enrolled patients and divided them into two groups, CP-dominant group and FFP-dominant group, according to the ratio of FFP to CP used in the replacement fluid. There were no significant differences in demographic and clinicopathological characteristics (shown in Table 1) between the two groups, except for INR. The distributions of PLASMIC and Bentley scores were similar in the two groups. Other therapeutics used, such as steroid, vincristine, cyclophosphamide, IVIg, and rituximab, were all similar in the two groups.

First, we analyzed the effects of the CP-dominant and FFP-dominant replacement fluids used on the early response to TPE in patients with TTP. With regard to the number of TPE procedures needed to achieve complete response, a higher number of TPE procedures was required in the FFP-dominant group than in the CP-dominant group. With respect to the time needed to achieve complete response, more time was needed in the FFP-dominant group than in the CP-dominant group. We hypothesized that a lower concentration of vWF in the CP-dominant replacement fluid may contribute to this result, since higher concentrations of vWF accelerate the formation of platelet thrombus in plasma [20].

Then, we analyzed the effect of the CP-dominant and FFP-dominant replacement fluid on the survival of patients with TTP. However, no significant difference was observed in the overall survival between the two groups.

In conclusion, our data showed that the CP-dominant replacement fluid was superior to the FFP-dominant replacement fluid in early response to TPE in patients with TTP, but did not have an impact on the patients' long-term survival. However, there are some limitations to our study. Since TTP is a rare disease, the limited sample size may restrict the validity of our results. Although the CP-dominant replacement fluid was a better choice, the difference in the FFP-to-CP ratio in each group makes it impossible to provide a precise ratio for the FFP and CP combination. Therefore, we believe that a prospective, multicenter, large-sample clinical trial is needed for further studies.

## Figures and Tables

**Table 1:** Demographic and clinicopathological characteristics of the enrolled patients.

Character	CP dominant	FFP dominant	P value
Number	11	16	
FFP/CP ratio	0.79 ± 0.04	1.43 ± 0.15	0.0015
Age(years)	48.00 ± 2.84	51.56 ± 4.21	0.53
Gender			1.00
male	5	8	
female	6	8	
PLASMIC score			0.39
5	1	0	
6	8	11	
7	2	5	
Bentley score			0.43
20-30	8	8	
>30	3	8	
Platelet count (×10^9^/L)	14.45 ± 2.30	14.63 ± 1.59	0.95
Uncorrected reticulocyte (%)	14.29 ± 1.82	12.57 ± 1.58	0.49
MCV (fL)	93.98 ± 3.67	95.75 ± 2.23	0.67
INR	1.07 ± 0.02	0.97 ± 0.03	0.04
Creatinine (*μ*mol/L)	86.90 ± 11.94	76.59 ± 4.91	0.38
Indirect bilirubin (*μ*mol/L)	29.25 ± 6.52	34.16 ± 5.80	0.58
Active cancer	1(9.09%)	1(6.25%)	1.00
D-dimer (ug/ml)	2.17 ± 0.44	2.21 ± 0.48	0.96
Procedures to complete response	4.00 ± 0.92	8.23 ± 1.63*∗*	0.04
Time to complete response (days)	5.82 ± 1.39	14.08 ± 3.32*∗*	0.04
Steroid use (%)	11(100%)	16(100%)	1.00
Vincristine use (%)	1(9.09%)	0(0%)	0.41
Cyclophosphamide use (%)	3(27.27%)	6(37.5%)	0.69
IVIg use (%)	4(36.36%)	10(62.5%)	0.25
Rituximab use (%)	1((9.09%)	2(12.5%)	1.00

IVIg: intravenous immunoglobulin; MCV: mean corpuscular volume; INR: international normalized ratio.

**Table 2:** Components of PLASMIC and Bentley score of the enrolled patients.

Patient Number	FFP/CP ratio	Platelet count (×10^9^/L)	Indirect bilirubin (*μ*mol/L)	Uncorrected reticulocyte (%)	Active cancer	MCV (fL)	INR	Creatinine (*μ*mol/L)	D-dimer (*μ*g/ml)	PLASMIC score	Bentley score
1	0.61	8	22.7	21.63	NO	108.6	1.04	89	2.52	6	21
2	0.88	20	17.5	16.59	NO	101.1	1.05	62	2.1	6	21
3*∗*#	1.86	6	65.1	22.29	NO	108	1.02	68	3.12	6	41.5
4	0.73	29	16.7	18.72	NO	94.4	1.13	64.3	1.65	6	21
5	0.93	9	23.5	14.83	NO	86.2	1.2	84	1.18	7	21
6#	1.48	28	16.1	16.39	NO	109.3	1.01	67	2.13	6	21
7	0.85	6	28.1	19.74	NO	104	1.1	66	2.09	6	41.5
8*∗*	0.72	16	12.2	10	NO	95	1.04	39	0.65	6	21
9	1.14	12	80.4	16.73	NO	107.3	0.73	101	2.22	6	41.5
10	1.11	16	23.3	20.21	NO	99	0.99	62	0.8	6	21
11	0.94	11	82.3	4.84	Thyroid	71.7	1.18	178	6.21	5	20
12	1.40	18	18.6	9.76	NO	96.5	0.81	81	1.47	6	21
13*∗*	1.81	6	73	6.55	NO	84.1	0.98	86	1.84	7	41.5
14	0.56	6	49.7	19.32	NO	108.8	0.97	66.8	2.42	6	41.5
15	1.21	13	31.5	13.48	NO	100.6	0.98	68.4	3.76	6	41.5
16	0.73	21	17.5	4.83	NO	76.3	0.92	79	1.35	7	21
17	3.45	11	19.5	19.47	NO	86.4	0.72	108	2.97	7	21
18#	1.12	18	17	4.22	esophagus	88.3	0.86	67	0.99	6	21
19	1.08	11	62	4.71	NO	87.2	1.03	92	1.08	7	41.5
20	1.28	10	38.4	8.94	NO	102.2	1.11	52	0.66	6	41.5
21	1.25	13	16.7	16.8	NO	106	1.09	74	0.89	6	21
22	1.17	24	36.6	7.42	NO	89.4	1.17	95	3.61	7	41.5
23*∗*	1.28	21	12.3	11.32	NO	92.5	1.04	104	1.07	6	21
24	1.17	19	8.9	3.4	NO	85.2	0.92	40	0.5	7	21
25	0.87	11	43.3	17.22	NO	90.4	1.07	85.8	1.68	6	41.5
26*∗*	1.16	8	27.2	19.43	NO	90	1.09	60	8.22	6	31.5
27	0.89	22	8.3	9.42	NO	97.3	1.05	142	2.01	6	21

MCV: mean corpuscular volume; INR: international normalized ratio; *∗*: patients who were evaluated for ADAMTS13 activity; #: patients who did not achieve complete remission before therapeutic plasma exchange ceased.

## Data Availability

Some data of the demographic and clinicopathological characteristics and survival of TTP patients used to support the findings of this study are restricted by the Ethics Committee of Fujian Medical University Union Hospital in order to protect patients' privacy. Other data used to support the findings of this study are included within the article. Data are available from the corresponding author (Haobo Huang: huanghaobo1981@163.com; Liping Fan: fanliping1982@163.com) for researchers who meet the criteria for access to confidential data.
